# Phenotypically anchored transcriptomics across diverse agrichemicals reveals conserved pathways and unique gene expression signatures in zebrafish

**DOI:** 10.3389/ftox.2025.1675060

**Published:** 2025-10-17

**Authors:** Lindsey St. Mary, Ryan McClure, Lisa Truong, Steven J. Carrell, Katrina M. Waters, Robyn L. Tanguay

**Affiliations:** ^1^ Sinnhuber Aquatic Research Laboratory, Environmental and Molecular Toxicology Department, College of Agricultural Sciences, Oregon State University, Corvallis, OR, United States; ^2^ Pacific Northwest National Laboratory, Biological Sciences Division, Richland, WA, United States

**Keywords:** zebrafish, transcriptomic profiling, agrichemical, carbosulfan, chlordane, modes of action

## Abstract

Agrichemicals such as herbicides, fungicides, insecticides, and biocides are widely used in agriculture, yet some are associated with adverse effects in humans and the environment. While many of these chemicals have been extensively studied *in vitro* and are included in the EPA’s ToxCast program, comprehensive *in vivo* comparisons using RNA sequencing across structurally diverse agrichemicals, in a single screening platform, are lacking. In this study, we examined structurally diverse agrichemicals found in the U.S. Environmental Protection Agency’s (EPA) Toxcast Phase I and II library by statically exposing early life stage zebrafish at 6 h post fertilization (hpf) until 120 hpf at concentrations ranging from 0.25 to 100 µM. Morphological outcomes were assessed at 120 hpf across 10 endpoints, including yolk sac edema, craniofacial malformations, and axis abnormalities. Chemicals that produced robust concentration-response relationships were selected for transcriptomic profiling. For transcriptomic analysis, zebrafish were statically exposed to each chemical and sampled at 48 hpf, prior to the onset of morphological effects observed at 120 hpf. Differential expression analysis identified between 0 and 4,538 differentially expressed genes (DEGs) per chemical, with no clear correlation to morphological severity. Both DEG and co-expression network analyses revealed chemical-specific expression patterns that converged on shared biological pathways, including neurodevelopment and cytoskeletal organization. Key regulatory genes such as *mylpfa* and *krt4* were identified within co-expression modules, suggesting their potential role in conserved toxicity mechanisms. Semantic similarity analysis of enriched gene ontology (GO) terms, when compared to existing datasets, highlighted gaps in the annotation of neurodevelopmental processes, indicating that some *in vivo* effects may not be fully captured by current curated resources. The results provide new insights into the modes of action of diverse agrichemicals and establish a framework for understanding how agrichemical structure relates to biological function in a vertebrate model.

## 1 Introduction

The agrichemical industry plays a pivotal role in supporting global population growth (Costa, 1987). Pesticides are the most well-known agrichemicals but also the most controversial (Aktar et al., 2009). According to the Pesticide Action Network (PAN), there are more than 17,000 currently on the market, with 800 registered for use in the United States (Schwingl et al., 2021). Pesticides include insecticides, fungicides, herbicides, and biocides. While they are primarily associated with the agrichemical industry, they are also utilized in public health to control disease-carrying insect populations (Ahmad et al., 2024). Researchers study pesticides intensively because their toxic effects often extend beyond target organisms, posing potential hazards to humans and other animals.

Pesticides are a structurally diverse group of chemicals that include organochlorines, organophosphates, pyrethroids, triazines, and azoles, among many others (Hassaan and El Nemr, 2020). Structural diversity arose from the need to target a wide range of pests effectively and to stay ahead of the development of resistance in target organisms. High pesticide diversity means that many different types of pesticides are used, each affecting organisms in different ways. As a result, a wide range of biological pathways can be disrupted, leading to a variety of toxic effects in exposed organisms. A common mechanism is neurological impairment in insects, which, due to biological conservation, can cause both acute and developmental neurotoxicity in humans and animals (Abreu-Villaça and Levin, 2017; Yang et al., 2023). Many pesticides induce developmental neurotoxic effects *in vitro* and *in vivo* (Abreu-Villaça and Levin, 2017). However, comprehensive transcriptomic data are lacking, particularly for whole-organism responses during development. Among the 23,492 PubMed manuscripts related to the pesticides investigated in this study, only 1,601 contained transcriptomic datasets submitted to GEO. Of those, 895 (56%) were *in vitro* studies while 706 (44%) were *in vivo* datasets. Notably, only 154 of the 706 *in vivo* studies examined target organism responses, representing only 19 pesticides (Supplementary Table S1). Transcriptomic analysis offers mechanistic and system-wide insight with a sensitivity that surpasses traditional morphological endpoints. Morphology-anchored *in vivo* transcriptomics deepens understanding of chemical effects and supports 21st-century toxicology goals to reduce animal use by generating predictive data sets.

Rodent model studies are expensive, and questions remain regarding which organs are most relevant to human hazard assessment and how well the results translate to human responses. While *in vitro* systems offer scalable results, they intrinsically lack the biological complexity necessary to capture multi-organ interactions, metabolic processes, and systemic responses that occur in whole organisms during development.

To address this gap while adhering to the 3Rs principles (Replacement, Reduction, and Refinement), we employed a transcriptomics approach using developmental zebrafish (*Danio rerio*) as an integrated, new approach method (NAM) that avoids the use of mammals in research while providing the biological complexity needed to examine multiple structures and assess biological responses that cannot be captured *in vitro*.

Zebrafish share substantial genetic and physiological homology with humans, including conserved neurological pathways (Sakai et al., 2018; Howe et al., 2013). Their optical transparency throughout development enables real-time observation of growth and detection of adverse morphological abnormalities (Rericha et al., 2024). Unlike *in vitro* models, zebrafish maintain intact organ systems, tissue-tissue interactions, and dynamic physiological processes that are essential for understanding agrichemical toxicity. Furthermore, development is a highly sensitive life stage when essentially all biological pathways are active at some point.

**FIGURE 1:**
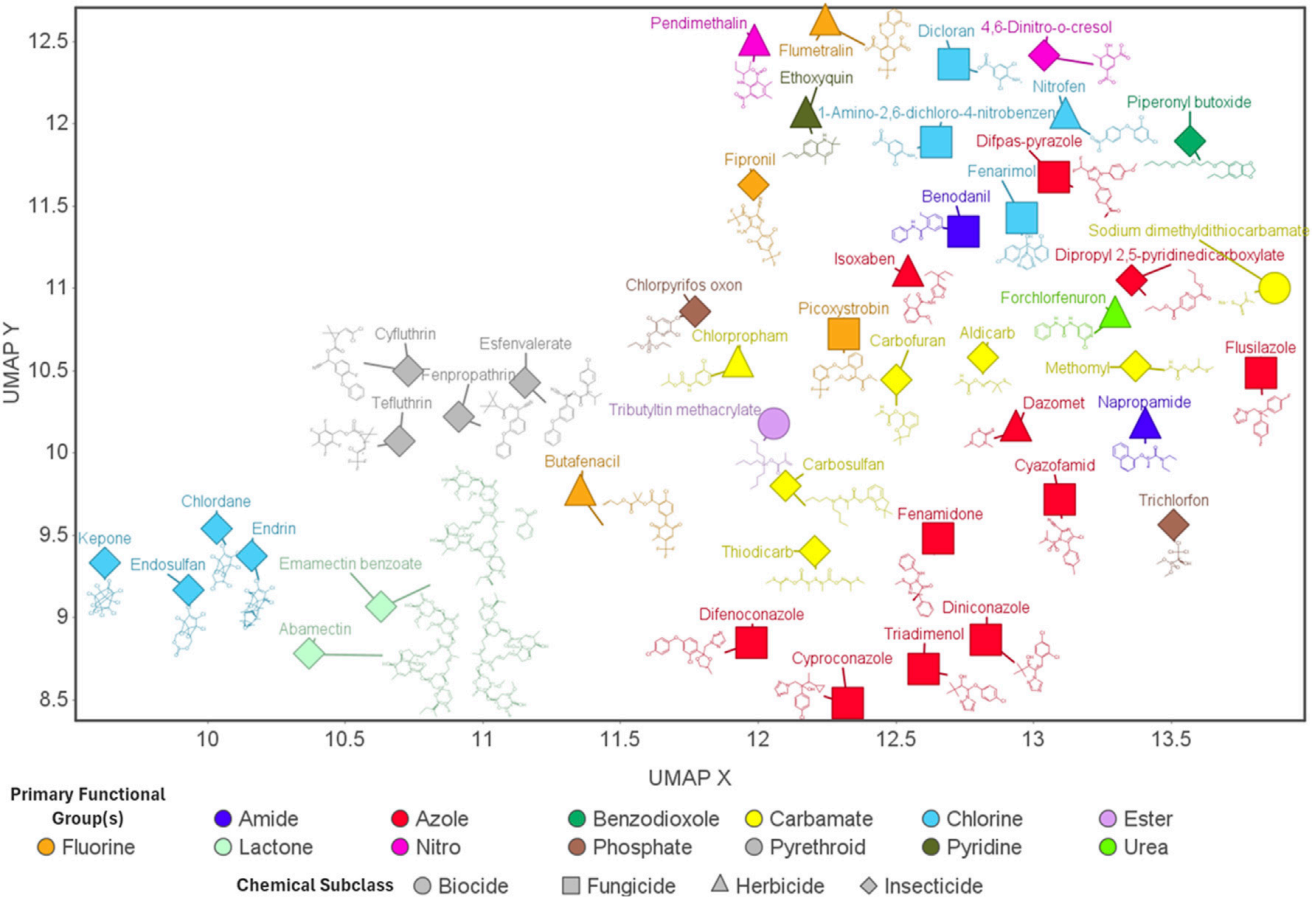
Agrichemicals: Essential Roles and Structural Diversity. UMAP (uniform manifold approximation and projection) visualization of all 45 agrichemicals investigated in this study. Each point represents an individual chemical, with shape denoting the chemical subclass (intended purpose classification) and color indicating the primary functional group. Chemical structures are displayed adjacent to their corresponding data points. This visualization highlights the diversity of chemical structures across agrichemical subclasses, as well as within the same subclass.

We screened agrichemical bioactivity in developing zebrafish to link transcriptome-wide changes to phenotypic outcomes. From this screen, 45 structurally diverse agrichemicals that exhibited robust concentration-response relationships were selected for transcriptomic analysis (Figure 1). Developmental exposure to these chemicals produced unique transcriptomic profiles at 48 hpf, indicating that structural diversity translated to distinct molecular-level responses. Gene ontology (GO) enrichment combined with gene and chemical co-expression analyses of transcriptomic responses revealed convergence on key biological processes, regardless of structural similarity or subclass (intended purpose classification). These findings underscore the importance of system-level approaches for evaluating agrichemical toxicity and demonstrate the value of integrating transcriptomic and phenotypic data to better understand how chemical structure influences biological activity during development.

## 2 Materials and methods

### 2.1 Zebrafish husbandry

All protocols were approved by Oregon State University’s Institutional Animal Care and Use Committee (IACUC; ACUP 2024-0510). Tropical 5D wild-type zebrafish were bred and maintained at 28 °C under 14:10 h light/dark cycle at Sinnhuber Aquatic Research Laboratory (SARL), Oregon State University. Fish were housed at densities of 500 fish per 50-gallon tank in a recirculating system supplemented with Instance Ocean salts. Routine health monitoring and feeding with age-appropriate Zebrafeed (Sparos) occurred 2–3 times daily, as previously described by Barton et al. (2016). Embryos were collected between 8:00 and 9:00 a.m., following light onset, using custom spawning funnels placed in tanks the night prior. Only fertilized embryos of high quality and matched developmental stage were selected (Kimmel et al., 1995). Embryos were kept at 28 °C in embryo medium (EM) containing 15 mM NaCl, 0.5 mM KCL, 1 mM MgSO_4_, 0.15 mM KH_2_PO_4_, 0.05 mM Na_2_HPO_4_, and 0.7 mM NaHCO_3_ (Westerfield, 2000).

### 2.2 Chemical selection and experiment overview

The agrichemicals chosen for the developmental toxicity screening consisted of a subset of pesticides found within the U.S. EPA’s ToxCast Phase I and II chemical library that had previously demonstrated developmental toxicity in zebrafish (Truong et al., 2014). We re-evaluated a subset of chemicals from Truong et al. (2014) using an updated screening approach (Table 1). Concentration-response assays were conducted to estimate the concentrations at which 80% of animals exhibited malformations (EC_80_) at 120 hpf. To capture early transcriptional responses that precede the observed morphological responses at 120 hpf, animals were collected at 48 hpf. A small number of chemicals caused morphological effects and mortality at 48 hpf, and several of these showed clear, dose-dependent responses. To accommodate, we used an EC range of 13–100, successfully attained for all chemicals, and used EC_80_ where possible to capture biologically relevant responses below overt toxicity thresholds at 48 hpf (Rericha et al., 2024). This approach ensured that we performed transcriptomic profiling at concentrations that clearly caused developmental toxicity. The experimental design is illustrated in Supplementary Figure S1.

**TABLE 1:** Agrichemicals evaluated in this study are listed along with their CAS Registry Numbers (CASRN), chemical subclasses, nominal exposure concentrations, and the final effective concentrations (EC) used for transcriptomic analysis where applicable.

Chemical	CASRN	Subclass	Concentration	EC value
4,6-Dinitro-o-cresol	534–52–1	Insecticide	24.5	13
Thiodicarb	59669–26–0	Insecticide	4.5	25
Dipropyl 2,5-pyridinedicarboxylate	136–45–8	Insecticide	6	31
Carbofuran	1563–66–2	Insecticide	49	38
Methomyl	16752–77–5	Insecticide	20	38
Trichlorfon	52–68–6	Insecticide	68	38
Chlorpyrifos oxon	5598–15–2	Insecticide	5.95	50
Piperonyl butoxide	51–03–6	Insecticide	14.5	50
Benodanil	15310–01–7	Fungicide	8	56
Ethoxyquin	91–53–2	Herbicide	45	57
Diniconazole	83657–24–3	Fungicide	10	69
1-Amino-2,6-dichloro-4-nitrobenzene	99–30–9	Fungicide	25	75
Fenamidone	161326–34–7	Fungicide	60	75
Dazomet	533–74–4	Herbicide	20	81
Fenpropathrin	39515–41–8	Insecticide	1.5	81
Forchlorfenuron	68157–60–8	Herbicide	18.5	81
Carbosulfan	55285–14–8	Insecticide	25	88
Chlordane	57–74–9	Insecticide	64	88
Chlorpropham	101–21–3	Herbicide	58	88
Esfenvalerate	66230–04–4	Insecticide	2.5	88
Difenoconazole	119446–68–3	Fungicide	9	94
Difpas-pyrazole	151506–44–4	Fungicide	60	94
Napropamide	15299–99–7	Herbicide	72	94
Picoxystrobin	117428–22–5	Fungicide	0.75	94
Tributyltin methacrylate	2155–70–6	Biocide	0.25	94
Abamectin	71751–41–2	Insecticide	4.5	100
Aldicarb	116–06–3	Insecticide	15	100
Butafenacil	134605–64–4	Herbicide	19.5	100
Cyazofamid	120116–88–3	Fungicide	16	100
Cyfluthrin	68359–37–5	Insecticide	0.62	100
Cyproconazole	94361–06–5	Fungicide	55	100
Emamectin benzoate	155569–91–8	Insecticide	4.5	100
Endosulfan	115–29–7	Insecticide	3.25	100
Endrin	72–20–8	Insecticide	0.6	100
Fenarimol	60168–88–9	Fungicide	30	100
Fipronil	120068–37–3	Insecticide	10	100
Flumetralin	62924–70–3	Herbicide	30	100
Flusilazole	85509–19–9	Fungicide	15	100
Isoxaben	82558–50–7	Herbicide	60	100
Kepone	143–50–0	Insecticide	5	100
Nitrofen	1836–75–5	Herbicide	20	100
Pendimethalin	40487–42–1	Herbicide	38	100
Sodium dimethyldithiocarbamate	128–04–1	Biocide	10	100
Tefluthrin	79538–32–2	Insecticide	10	100
Triadimenol	55219–65–3	Fungicide	59	100

### 2.3 Chemical exposure and developmental toxicity screening of transcriptomic samples

At 4 hpf, embryos were enzymatically dechorionated using pronase, as previously described (Mandrell et al., 2012). Dechorionated embryos were singulated into 96-well U-bottom plates (Falcon, Product no. 353227) containing 100 µL of embryo medium (EM) via robotic placement (Mandrell et al., 2012). At 6 hpf, chemicals were dispensed using an HP D300 digital dispenser to achieve the target nominal concentrations while also maintaining a 0.5% DMSO concentration across all exposures.

Static chemical exposures from 6 to 120 hpf were conducted in 96-well plates to estimate EC_80_. Initial range-finding assays included four concentrations (including controls) with one row per concentration (n = 12) and two chemicals per plate, to identify concentrations eliciting adverse morphological effects. Based on these results, concentration–response assays were performed using eight concentrations (including controls), with one row per concentration (n = 12) and a single chemical per plate, to refine the effective concentration range and enable EC_80_ estimation via logistic regression modeling. A definitive assay was conducted using the selected EC concentration across columns (n = 16) to confirm morphological responses by 120 hpf and verify the absence of mortality and morphological abnormalities at 48 hpf.

Exposure concentrations for transcriptomic sampling ranged from 0.25 to 100 µM. At 120 hpf, 10 morphological endpoints were assessed and the percent incidence recorded (Supplementary Table S2). A cumulative measure, termed “any effect”, was the incidence of abnormality in any morphological endpoint. Logistic regression modeling of the “any effect” data was used to generate concentration-response curves to estimate EC_80_ for each chemical. For exposures destined for transcriptomics, embryos were exposed to the confirmed EC_13_-EC_100_ concentration of each chemical beginning at 6 hpf, and samples were collected at 48 hpf for subsequent RNA extraction (n = 5 pools of 8 embryos), prior to the onset of morphological effects. Holdback plates (n = 16 per chemical) were used to assess and confirm phenotypic outcomes. Collections occurred over 23 different days which resulted in 23 different day match controls.

### 2.4 Transcriptomic sample preparation and data processing

#### 2.4.1 RNA collection and isolation

Pooled embryos were placed in 1.5 mL twist cap microtubes (NextAdvance, TUBE1R5-S) and placed on ice for euthanasia. Excess water was removed and 200 µL of 1X RNAshield (Zymo Research, 76,020-420) was added to the tube after animals remained on ice for 15 min. Samples remained at room temperature for up to 15 min and then stored at −20 °C until RNA isolation. Five pools of 8 embryos were collected for each exposure and respective controls.

For RNA isolation, sample tubes were removed from −20 °C and incubated at 32 °C for 30 min to ensure complete thawing. 100 μL of 0.5 mm zirconium silicate beads (BioSpec Products, 11079105z) were added, and samples were homogenized in the Bullet Blender Storm Pro (NextAdvance, BT24MB-50759) at speed 10, for 5 min. Samples were spun down for 5 min at 12,000 x g, afterwards 250 µL of supernatant from each tube was aliquoted twice (for a total of 500 µL) into two new sample plates. These plates were then processed using the KingFisher Apex system for automated RNA extraction using Zymo Research’s Quick RNA Magbead Kit (R2133) and eluted into 50 µL RNAse/DNAse free water. Sample quality was checked on the Agilent Tapestation 4,200 and quantity was assessed using ThermoFisher’s QuantIT assay. Only samples with RIN values >8 proceeded to RNA sequencing.

#### 2.4.2 RNA sequencing and data processing

mRNA sequencing was performed on four biological replicates of the five collected for each treatment. The samples underwent PrepX Robotic RNA PolyA enrichment combined with NEBNext UltraII Directional RNA Library Prep/Illumina Stranded mRNA library preparation. Samples were split between three P3 flow cells (1.2 billion reads per run) and a P2 (400 million reads per run), and samples were sequenced using an Illumina NextSeq 2,000 (100 bp single end) resulting in 20 million read counts for each sample. All raw and processed transcriptomic data generated in this study are available in the NCBI Gene Expression Omnibus (GEO) under accession number GSE303009.

Raw fastq files were examined with FastQC and trimmed using Trimmomatic. Any read with a quality Phred score of <25 was trimmed and any adaptor sequences were trimmed as well. Trimmed fastq files were then aligned to the v11 *Danio rerio* genome (RefSeq GCF_000002035.6). Alignment was done using the STAR aligner with the following arguments--outFilterType BySJout, --outFilterMultimapNmax 20, --readFilesCommand zcat, --alignSJoverhangMin 8, --alignSJDBoverhangMin 1, --outFilterMismatchNmax 999, --outFilterMismatchNoverLmax 0.6, --alignIntronMin 20, --alignIntronMax 1000000, --alignMatesGapMax 1000000, --outSAMattributes NH HI NM MD, --outSAMtype SAM (Dobin et al., 2013). Once aligned, resulting SAM files were converted to count files using HTSeq using the following arguments -f sam -a 10 -t gene -i gene_id (Anders et al., 2015). Raw count files were then normalized using DESeq2 (Love et al., 2014). DESeq2 was also used for differential gene expression analysis. To account for potential variability across collection days, we used day matched vehicle controls for each of the 23 distinct RNA-collection days. We concatenated the gene count data from all control samples (n = 23) to form a single global control dataset. This global control served as the normalization reference for all agrichemical groups, allowing consistent comparisons of gene expression change across samples. The criteria for defining DEGs was set to any log2fold changes with p-value ≤0.05, without applying a log2fold cutoff.

#### 2.4.3 Gene coexpression network analysis

To infer a gene co-expression network all normalized gene expression data was used in conjunction with GENIE3, a random forest approach that produces a matrix of co-expression values for any gene pair in the dataset (Huynh-Thu et al., 2010). For each gene it builds a regression model using expression values of all genes to predict how genes are co-expressed. Once a co-expression matrix was inferred we selected several potential co-expression value cutoffs to infer a network with sufficient genes included but also with clear structure, allowing us to better analyze how genes are related by expression. A cutoff value of 0.00923 was selected which led to a network of 735 genes and 1716 edges between the genes. This network was viewed using Cytoscape and this same program was used to calculate centrality values of each node in the network (Shannon et al., 2003). We performed centrality analysis on all genes in the coexpression network using both betweenness and degree metrics. Betweenness measures how often a node serves as a bridge on the shortest paths between other nodes, highlighting those that have a strong influence on information flow within the network. A node with high betweenness connects different parts of the network. Degree, on the other hand, counts the number of direct connections a node has, helping identify genes that are highly connected and influential within the network. Together this offers a more comprehensive understanding of node (gene) influence and identified key regulatory genes (McDermott, et al., 2009). Module detection was done using the fastgreedy. community function in the igraph package in R (Csardi and Nepusz, 2006).

Functional enrichment analysis was performed on each gene coexpression module using g:Profiler, querying all available annotation sources to maximize biological coverage and identify overarching functional themes.

#### 2.4.4 Chemical coexpression network analysis

To infer a network linking chemicals rather than genes we used the same normalized gene expression data as above. Data were essentially transposed so that rows were now chemicals and columns were genes. We again used GENIE3 (with a co-expression cutoff value of 0.0787 to define an edge resulting in 46 nodes, 45 chemicals and 1 DMSO control, and 144 edges). We also used Cytoscape to view the network and determine centrality values.

#### 2.4.5 CTD enrichment comparisons

Chemical CAS registry numbers (CASRNs) were submitted to the Comparative Toxicogenomics Database (CTD; https://ctdbase.org/tools/batchQuery.go) to retrieve enriched GO associations from existing data. Custom R script was written to cluster GO terms by semantic similarity using rrvgo R package, calculateSimMatrix () and reduceSimMatrix () for each ontology. We then joined the clustered GO terms back into original data and summarized the number of parent terms per chemical, per ontology, per semantic cluster.

## 3 Results

### 3.1 Structural diversity of agrichemicals that induced developmental toxicity in zebrafish

To characterize the structural diversity of the agrichemicals analyzed through transcriptomic profiling in this study, we performed UMAP (Uniform Manifold Approximation and Projection) dimensionality reduction using molecular fingerprints. The resulting plot, seen in Figure 1, reveals substantial chemical heterogeneity across and within agrichemical subclasses (intended purpose classification).

Subclasses such as pyrethroids exhibited tight clustering, consistent with their structural similarity, whereas others-particularly fungicides-were more broadly dispersed, reflecting greater structural diversity. Within the fungicide subclass, flusilazole and picoxystrobin were the most divergent, clustering more closely with carbamates from different subclasses. Notably, chemicals sharing the same functional group classification did not consistently cluster together, highlighting the chemical diversity beyond functional group classification. Although insecticides were the most represented subclass, many formed distinct clusters, and some - such as piperonyl butoxide and trichlorfon - were highly divergent from both their subclass and functional group.

### 3.2 Morphological profiles across agrichemicals

**FIGURE 2:**
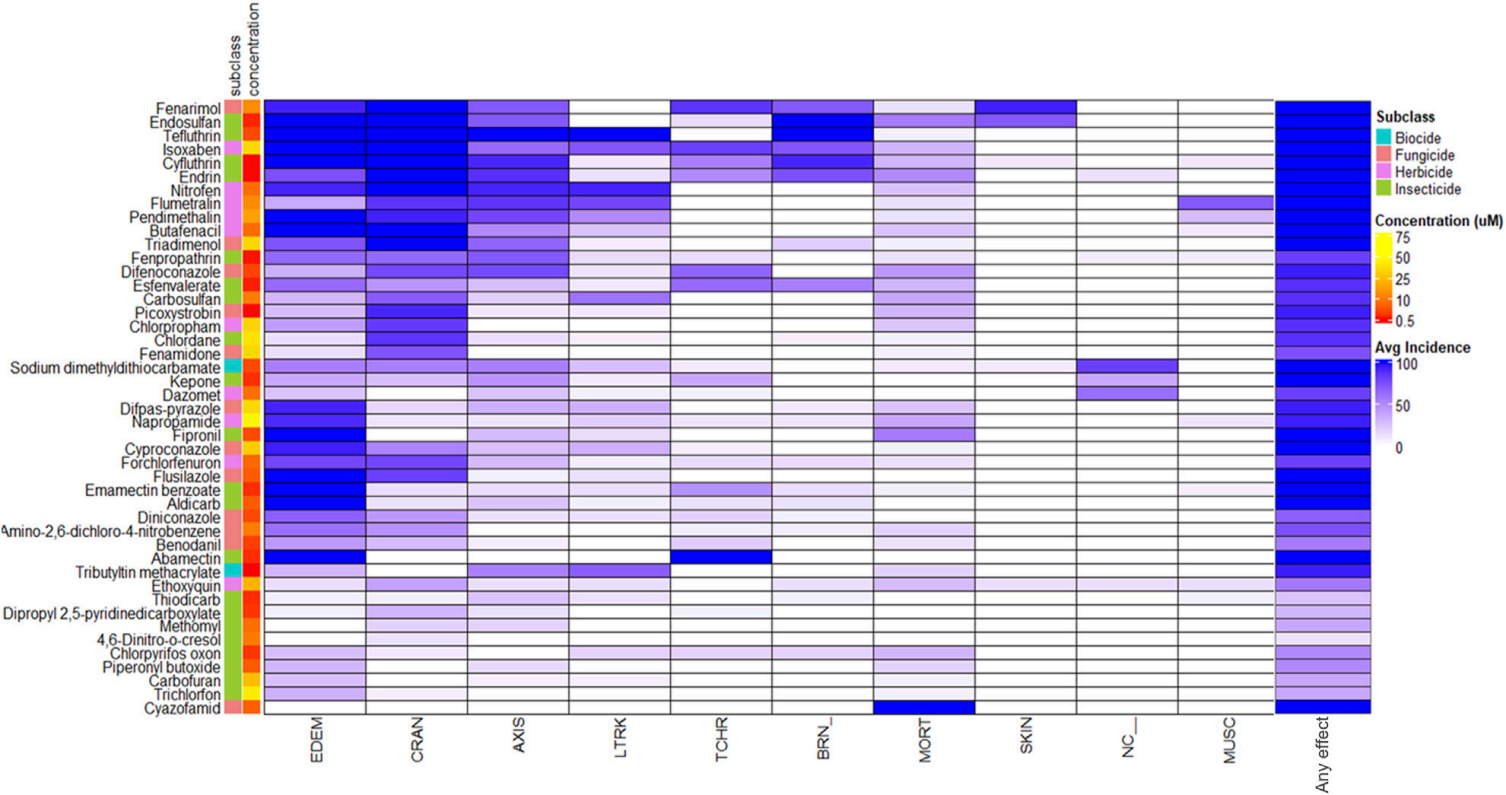
Morphological Profiles Across Agrichemicals. Heatmap of the morphology profiles across all the agrichemicals along with chemical names, subclass, and concentration annotated on the left side. Any effect was filtered out during the clustering but added to the end of the heatmap to show what the total % incidence for each of the agrichemicals. Chemicals were clustered according to morphological profile similarity. See Supplementary Table S2B for full descriptions of the morphological endpoints.

The 45 agrichemicals selected for transcriptomic analysis exhibited robust and reproducible concentration-responses at 120 hpf, and their corresponding morphological profiles are shown in Figure 2. No consistent pattern of malformation type was associated with the different chemicals or their subclasses. The concentrations at which phenotypic effects were observed ranged from 0.25 to 75 μM, indicating substantial variability in nominal concentration responses among the tested chemicals (Supplementary Table S2). For example, napropamide had an EC_94_ at 72 μM, while tributyltin methacrylate induced the same percent incidence (EC_94_) at 0.25 µM. Craniofacial abnormalities and edema were the most frequently observed morphological outcomes. Cyazofamid induced 100% mortality and was the only one that did so.

Thiodicarb, chlorpyrifos oxon, dipropyl 2,5-pyridinedicarboxylate, piperonyl butoxide, methomyl, 4,6-dinitro-o-cresol, and carbofuran induced ≤50% effect at the selected concentrations, as higher nominal concentrations caused excessive toxicity at 48 hpf, the designated collection time point for transcriptomics (Supplementary Table S2). No clear relationship was observed between the percent incidence and the corresponding exposure concentration across chemical subclasses.

### 3.3 Unique gene expression profiles showed functional convergence

**FIGURE 3:**
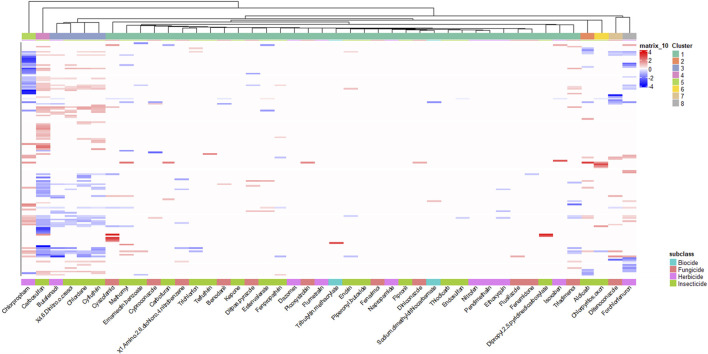
Unique Transcriptomic Signatures Across Agrichemicals. Heatmap of all differentially expressed genes (DEGs) identified across 44 agrichemicals (p-value ≤0.05). Abamectin, which had no DEGs, is excluded. Rows represented 8,546 unique DEGs, with no genes shared across all chemicals, though some overlap occurs among subsets. Chemical subclass annotations are shown at the bottom, and cluster assignments derived from hierarchical clustering are shown at the top. Chemicals were clustered using a custom R script based on dendrogram height differences to define major clusters.

To begin to understand the molecular mechanisms underlying these phenotypic responses, we conducted transcriptomic analysis at 48 hpf, prior to the manifestation of observable malformations. After identifying effective concentrations that induced phenotypic effects at 120 hpf for all the agrichemicals, we performed 48 hpf whole embryo transcriptomics. Differential expression analysis identified 8,546 DEGs in at least one agrichemical (Figure 3; Supplementary Table S3). Abamectin exposure produced no DEGs at 48hpf. Carbosulfan induced the highest number of DEGs (4,538). To identify patterns in transcriptomic responses, we used hierarchical clustering, which revealed eight distinct groups of chemicals. Most chemicals (34) grouped into Cluster 1, showing similar, but distinct expression profiles (Supplementary Figure S2). Six clusters consisted of single chemicals with highly divergent and unique expression signatures. Cluster 3 contained four chemicals (butafenacil, 4,6-dinitro-o-cresol, chlordane, and cyfluthrin) that shared 479 overlapping DEGs. Functional enrichment analysis of these 479 shared DEGs highlighted pathways such as *erbB* signaling and anatomical structure development (Supplementary Figure S3).

**FIGURE 4:**
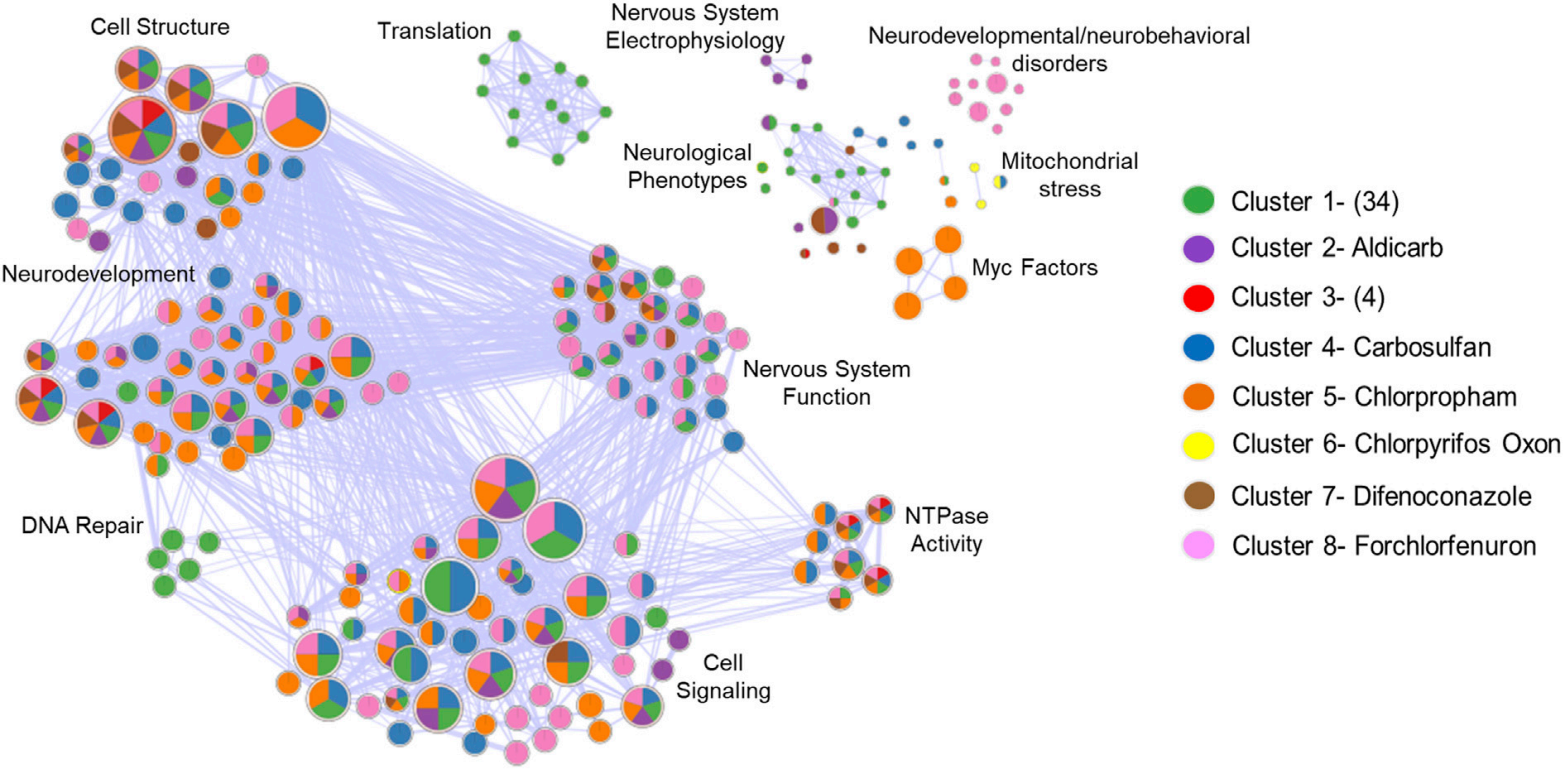
Diverse Transcripts Yield Overlapping Biological Themes. Enrichment map to visualize the GO terms and pathways across the 8 different DEG clusters identified in Figure 3 into a network to identify biological themes across the diverse gene sets. The network shows how genes in these clusters converge on biological processes, as well as those that don’t. Each node represents enriched biological terms, node size reflects gene set size which is the number of genes driving that GO term, pie chart colors correspond to DEG clusters identified in Figure 3 GO term overlap across DEG clusters. Functionally similar terms were grouped and manually annotated into broader biological processes (Cell Structure, Neurodevelopment, etc.).

We performed functional enrichment analysis across all eight clusters from Figure 3 to identify broader patterns. For Cluster 1, which had no genes shared across all chemicals within the cluster, we analyzed all DEGs (n = 3,208) from the cluster. The analysis showed that seven out of eight clusters were enriched for terms related to membrane structure, anatomical structure development, and NTPase activity (Figure 4). This analysis identified recurring enrichment of terms related to neurodevelopment (7/8 DEG clusters), nervous system function (6/8 DEG clusters), cellular structure (7/8 DEG clusters), and general developmental processes like NTPase activity and cell signaling (7/8 DEG clusters). Cluster-specific enrichment included neurodevelopmental and neurobehavioral disorder terms in Cluster 8 (forchlorfenuron) and nervous system electrophysiology in Cluster 2 (aldicarb). Overall, both shared and unique pathways were enriched across the clusters. Several disconnected terms in the enrichment network were driven by non-overlapping gene sets. These results highlighted convergence on core biological processes such as cellular stress, compensatory signaling, translation, and neurodevelopment, despite considerable transcriptomic heterogeneity.

### 3.4 Functional enrichment analysis revealed agrichemical effects and validated existing data

Enrichment analysis of agrichemicals with significant DEGs identified 726 GO terms (Supplementary Table S4). Ten chemicals (carbosulfan, isoxaben, benodanil, endosulfan, nitrofen, pendimethalin, flumetralin, dazomet, piperonyl butoxide, napropamide) did not lead to significant GO enrichment. To identify higher-order patterns, semantic similarity analysis grouped enriched terms into 45 parent terms (Supplementary Table S5). GTPase activator activity and nervous system development were the most frequent parent terms, each appearing 42 times across nine chemicals, with additional parent terms related to cytoskeletal organization and cellular processes. For example, 4,6-dinitro-o-cresol was consistently linked to nervous system development, while chlorpropham was uniquely enriched for *myc* transcription factor and cytoskeletal terms.

To validate these findings, we compared our GO terms to those in the Comparative Toxicogenomics Database (CTD). We identified 88 overlapping GO terms across nine chemicals, spanning cellular component (CC), biological process (BP), and molecular function (MF) categories (Supplementary Table S6). CTD contained 21,916 chemical-specific GO terms across 30 chemicals, 15 chemicals either lacked GO term annotations in CTD or could not be found using their CAS registry numbers (Supplementary Table S7). Among the overlapping terms, anatomical structure development and developmental process were the most frequently represented. Abamectin was associated with 757 GO terms in CTD, but no terms were identified in our study due to the absence of differentially expressed genes (DEGs) at the 48 hpf timepoint. Cyfluthrin had the highest number of overlapping GO terms, 59 in total, primarily associated with nervous system development, function and general developmental processes. A second semantic similarity analysis of the 88 overlapping GO terms produced 21 parent terms with cell differentiation identified as a shared parent term across aldicarb, cyfluthrin, difenoconazole, and endrin, though nervous system-related parent terms were not recovered (Supplementary Table S8).

A comparative analysis revealed a distinct emphasis between the datasets. CTD data were most enriched for BMP signaling (720 occurrences across 25 chemicals) and DNA binding (121 occurrences across 23 chemicals), whereas our data uniquely emphasized GTPase activity and nervous system development (Supplementary Table S9). Notably, CTD highlighted immune-related pathways more prominently, while our dataset revealed stronger enrichment for cell structure and cytoskeletal organization. Of the 726 chemical-specific terms identified in our study, 229 originated from non-GO sources (e.g., Human Phenotype Ontology (HP) and KEGG), while the remaining 409 terms were derived from GO categories (BP, MF, CC), indicating associations relative to the CTD.

### 3.5 Gene coexpression network reveals key regulators and distinct functional modules

**FIGURE 5:**
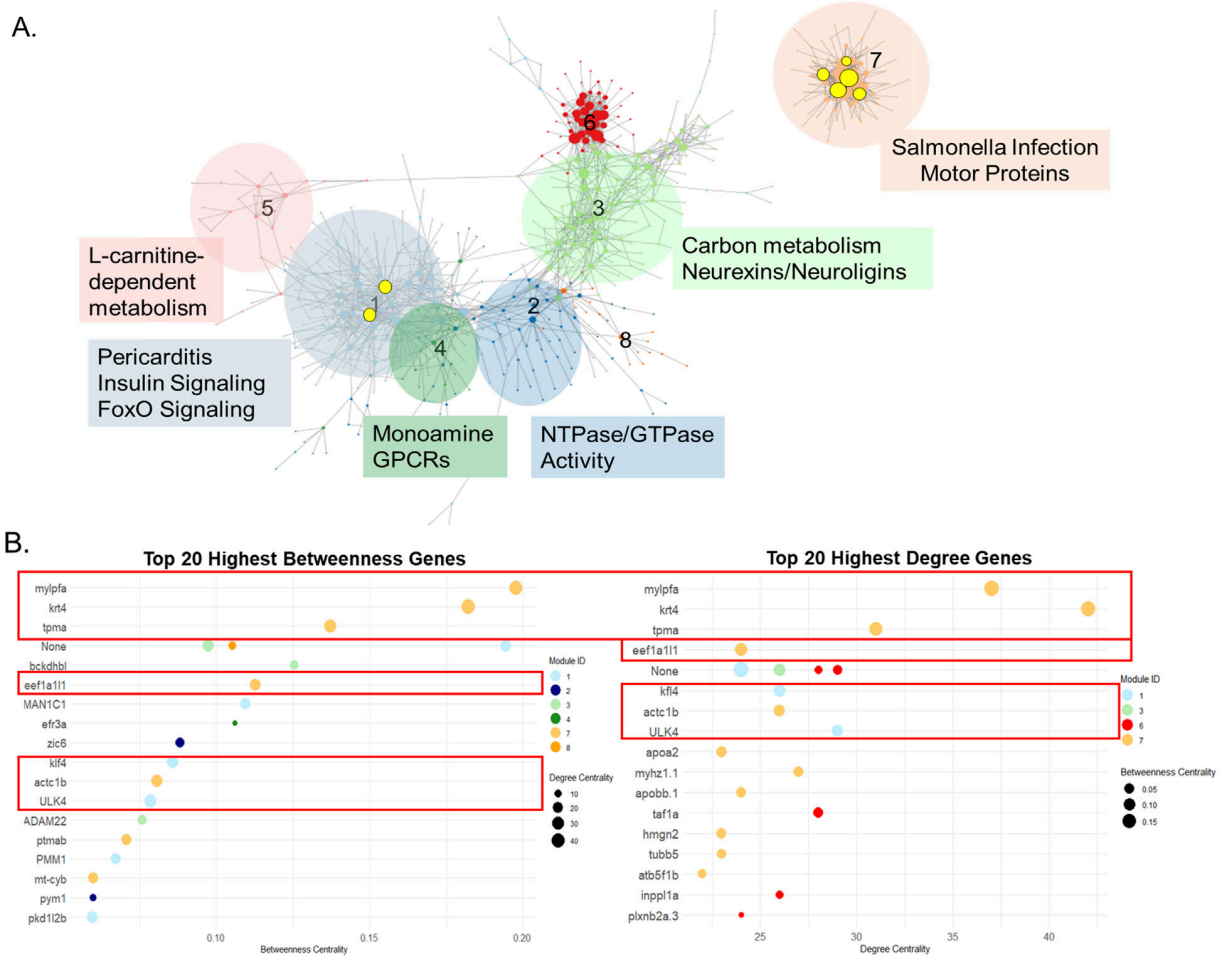
Centrality Analysis Revealed Biologically Relevant Regulatory Genes. **(A)** Gene coexpression network of all 45 chemicals, highly coexpressed genes were grouped into 8 modules. The size of the dot correlates with the degree, number of connections a single gene has to other genes. The black, unhighlighted text refers to the GO terms that resulted from functional enrichment analysis for the genes contained within each module. The yellow highlighted texts are the gene names of most central genes according to centrality metrics and the yellow circles are the associated genes. **(B)** Top 20 highest betweenness and degree genes found in the gene coexpression network. The red boxes indicate the same genes found between both metrics, which also appear highlighted in the coexpression network.

To identify regulatory relationships beyond individual DEGs, gene co-expression network analysis was performed across all samples. This analysis identified eight distinct modules, each representing genes with significantly correlated expression patterns (Figure 5A). Functional enrichment analysis of the genes within each module revealed distinct GO terms (Table 2). NTPase activity was the most significantly enriched GO term from module 2, while modules 3, 4, and 7 resulted in nervous system-related terms. Module 3 was specifically enriched for neurexin/neuroligin terms driven by two genes-*nlgn3a* and *nlgn1*. These genes also drove synapse and abnormal communication behavior terms in cluster 4 (carbosulfan) from the previous DEG analysis from Figure 3.

**TABLE 2:** Gene coexpression network module information which includes module ID, the number of nodes within each module, the module color, the enriched functions, and the adjusted p-value and functional enrichment score.

Module ID	# of nodes	Module color	Enriched function(s)	Adjusted p-value	Functional enrichment
1	209		FoxO signaling pathway	0.02	5.61
			Insulin signaling pathway	0.02	5.61
			Constrictive pericarditis	0.05	170.48
			Osteoarthritis of the elbow	0.05	170.48
			Generalized morning stiffness	0.05	170.48
			Flattened metacarpal heads	0.05	170.48
2	63		GTPase regulator activity	0.02	20.22
			nucleoside-triphosphatase regulator activity	0.02	20.22
			small GTPase-mediated signal transduction	0.03	26.96
3	137		Carbon metabolism	0.02	9.70
			Neurexins and neuroligins	0.04	81.89
4			Monoamine GPCRs	0.05	81.59
5	25		Effect of L carnitine on metabolism	0.05	81.59
6	48		-	-	-
7	81		Motor proteins	0.00	10.08
			Salmonella infection	0.00	4.75
8	18		-	-	-

Network centrality analysis identified seven central genes in the coexpression network based on centrality metrics (betweenness and degree) (Figure 5B). Functional enrichment analysis of these seven genes yielded only a single pathway - cardiac muscle contraction - driven by *tpma* and *actc1b*, suggesting limited functional overlap when considered as a group. However, module-specific analysis revealed more distinct functional roles. Five of the seven central genes were in module 7, which was enriched for cytoskeletal and host-response pathways (e.g., *salmonella* infection, motor protein activity). All five central genes (*mylpfa, krt4, tmpa, eef1a1l1, actc1b*) located in module 7 were associated with cytoskeletal organization, calcium signaling, or GTP-dependent protein synthesis-key pathways regulating cytoskeletal structure and function. The remaining two genes were in module 1, which was enriched for inflammatory and metabolic signaling pathways, including *foxO* signaling, insulin signaling, and pericarditis. The most central genes found in module 1, *klf4* and *ulk4*, were associated with LEF-1 transcription factor processes (lymphoid enhancer-binding factor 1). LEF-1 is a key regulator of *wnt* and beta-catenin signaling-pathways critical for development, cell proliferation, and differentiation.

### 3.6 Chemical coexpression network clustering highlighted transcriptomic groupings beyond subclass distinctions

**FIGURE 6:**
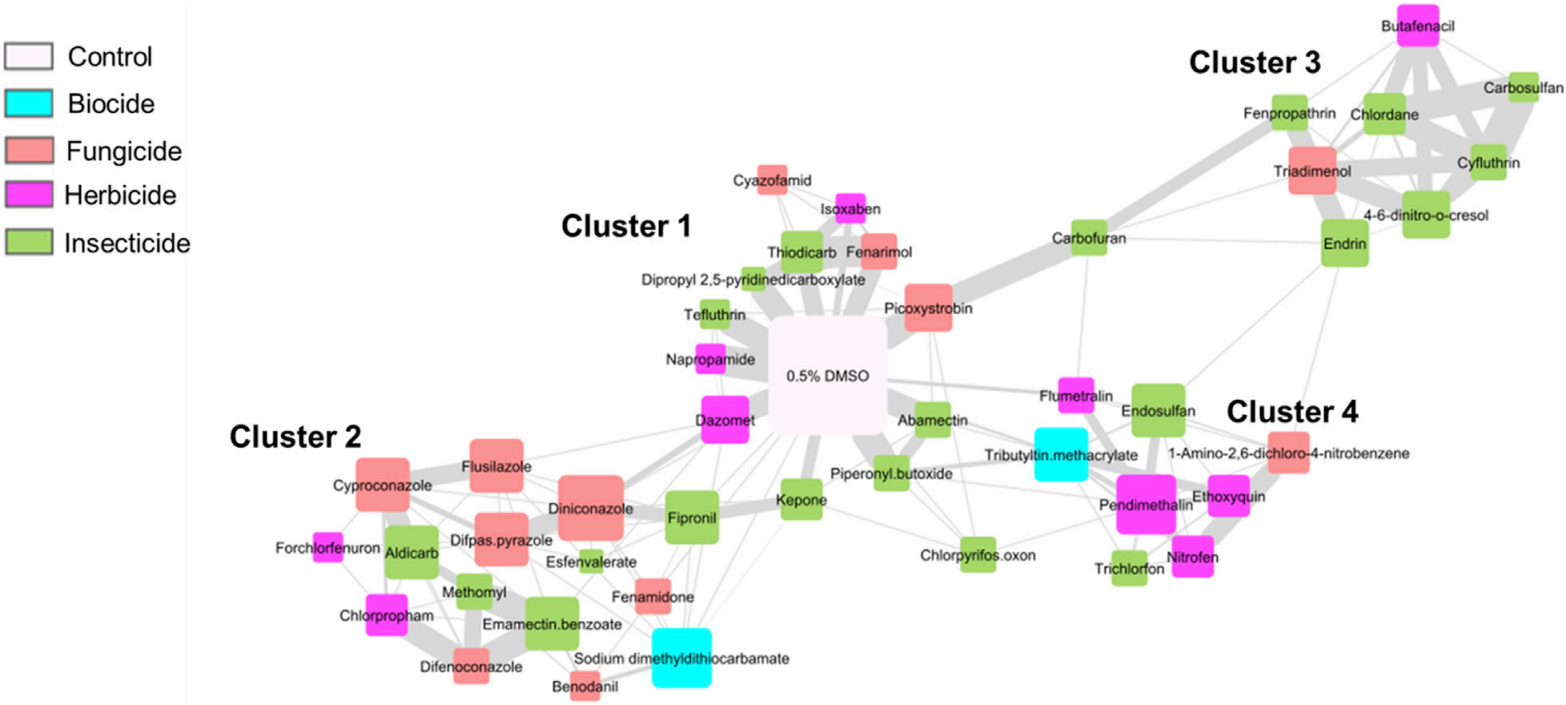
Chemical Coexpression Network Identified Chemical Clusters Based On Gene Expression Profiles. Chemical coexpression network of all 45 agrichemicals, grouped into 4 distinct clusters. Node size reflects degree (number of connections), and edge thickness indicates strength of coexpression (edge weight). Node colors correspond to chemical subclasses. The 0.5% DMSO control represents the global control, positioned at the center of the network (white node).

To complement gene coexpression analysis and to examine global transcriptomic similarities among agrichemicals, we constructed a chemical coexpression network from the same normalized gene expression data as was used for the gene co-expression network (Figure 6). Here, instead of genes linked based on their similar expression profiles across chemicals, chemicals were linked in a network based on similar expression patterns of the gene responses to the chemicals. This approach aimed to identify shared transcriptomic responses that might indicate common mechanisms of action across the tested agrichemicals.

The network analysis revealed four distinct chemical clusters with varying degrees of transcriptomic perturbation. Cluster 1 was closely associated with the DMSO controls, indicating minimal transcriptomic effects from these chemicals at this single developmental timepoint. Cluster 2 comprised 15 chemicals, with fungicides representing the most abundant subclass (seven chemicals). Cluster 3 included eight chemicals, with six of them being insecticides. Notably, four chemicals within Cluster 3—butafenacil, 4,6-dinitro-o-cresol, chlordane, and cyfluthrin—also exhibited a shared set of 479 DEGs in the hierarchical clustering analysis (Section 3.3; Figure 3), confirming their transcriptomic similarity across different analytical methods. Cluster 4 consisted of eight chemicals, half of which were herbicides.

Two chemicals - chlorpyrifos oxon and carbofuran - did not cluster with any group, due to their highly divergent expression profiles. These chemicals also demonstrated unique transcriptomic patterns in the DEG analysis and showed abnormal phenotypes in ≤50% of animals (50 and 37.5, respectively).

Chemical clustering patterns did not align strictly with chemical subclass designations. Each cluster resulted from chemicals in at least three of the four subclasses tested, indicating that transcriptomic similarity was not solely driven by chemical subclass but likely reflected shared biological responses or molecular targets.

## 4 Discussion

Our transcriptomic analysis revealed distinct and shared transcriptional responses to a diverse panel of developmentally toxic agrichemicals, offering insights into potential mechanisms of toxicity. The identification of 8,546 DEGs highlighted the sensitivity of early development to chemical exposure, though linking molecular changes to phenotypic outcomes proved more complex than expected.

### 4.1 Temporal dynamics of phenotypic anchoring and transcriptomic responses

For some chemicals there was no correlation between the percent incidence of malformation at 120 hpf and the number of DEGs identified at 48 hpf. For instance, endosulfan and forchlorfenuron induced morphological effects in 81.25% of exposed animals by 120 hpf but resulted in 7 and 1,872 DEGs at 48 hpf, respectively. This disconnect suggested that the molecular mechanisms driving developmental toxicity involved distinct temporal patterns and dose-response dynamics, aspects that were not adequately captured by measuring DEGs at a single timepoint (White et al., 2017). Several factors might have contributed to this phenomenon: differential chemical bioavailability and accumulation kinetics, metabolism, and the possibility that the molecular targets were not expressed until after the 48 hpf timepoint (Rericha et al., 2024). The selection of the 48 hpf timepoint was intended to target a developmental stage prior to the appearance of overt phenotypic abnormalities observed at 5 dpf, with the goal of identifying molecular events underlying the subsequent morphologies. Nonetheless, this time point may, in certain instances, precede critical developmental processes and thus limit detection of relevant molecular profiles. For instance, while most chemicals induced robust gene expression changes by 48 hpf, the absence of DEGs for abamectin at this timepoint suggested a later developmental window for its mechanism of action (MoA). This interpretation was supported by previous studies demonstrating abamectin-induced expression of apoptotic and inflammation-related genes in zebrafish at 96 hpf, and ossification-related genes at 14 days post fertilization (dpf) (Zhang et al., 2025; Wang et al., 2025). The delayed transcriptomic response pattern observed for several chemicals, particularly those grouped around the DMSO node in the chemical coexpression network in Figure 6, indicated that comprehensive toxicological assessments may require sampling across multiple developmental timepoints (Rericha et al., 2024).

In addition, gene expression data were generated using a single EC for each agrichemical. Although this approach facilitates comparative analysis, limiting sampling to one concentration could underrepresent the full molecular response space, particularly at lower or higher exposure concentrations where important molecular changes may emerge.

Finally, it is possible that some chemicals exert their effects through post-transcriptional mechanisms or by disrupting protein function directly rather than through gene expression changes. These findings highlight the benefits of integrating multiple endpoints and timepoints when characterizing chemical modes of action.

### 4.2 Transcriptomic clustering revealed functional overlap among structurally unrelated compounds

DEG clustering analysis revealed that most agrichemicals displayed highly divergent expression profiles, supporting chemical-specific modes of action. However, a subset of chemicals: butafenacil, 4,6-dinitro-o-cresol, chlordane, cyfluthrin, produced a coherent transcriptomic cluster, sharing a core set of DEGs enriched for processes like *erbB* signaling and anatomical structure development (Supplementary Figure S3). This shared profile was also reflected in our chemical coexpression network, which grouped these four compounds based on overall gene expression similarity despite their structural and mechanistic diversity. Among the three insecticides and one herbicide, they differed substantially in both chemical structure and their intended biological targets. Butafenacil targets protoporphyrinogen oxidase (PPO), essential for chlorophyll synthesis in plants and heme synthesis in animals; 4,6-dinitro-o-cresol uncouples oxidative phosphorylation; chlordane antagonizes GABA receptors; and cyfluthrin inhibits sodium channels (Leet et al., 2015; Atsdr, 2018; Dodmane and Koshlukova, 2024; Elyazar et al., 2011). While chlordane and cyfluthrin act directly on the nervous system - affecting both target and non-target organisms - butafenacil, through PPO inhibition, can disrupt heme synthesis and induce anemia in developing zebrafish. Similarly, 4,6-dinitro-o-cresol impairs mitochondrial ATP production, impacting energy metabolism across taxa (Leet et al., 2014). In our study, butafenacil’s impact on heme synthesis was captured by enrichment of metal ion binding terms, consistent with previous findings (Supplementary Table S3) (Leet et al., 2015). The intended target, alone, did not predict toxicological outcome, particularly during the sensitive period of embryonic development and highlighted the need for comprehensive mechanistic characterization across structurally diverse compounds.

### 4.3 Cytoskeletal disruption as a common downstream pathway

The convergence of multiple pesticides on motor protein-related biological processes was revealed through GO enrichment and gene coexpression network analysis. A key coexpression module (module 1) enriched for motor protein function and contained highly central genes involved in cytoskeletal organization and muscle morphogenesis (e.g., *krt4, tpma, actc1b, mylpfa*), suggesting that disruption of cytoskeletal dynamics represents a common biological disruption that is critical in neurodevelopment (Tang and Jin, 2018). This convergence was exemplified by two structurally related carbamates, carbofuran and chlorpropham, which produced remarkably similar biological signatures despite targeting different molecular pathways. While carbofuran inhibits acetylcholinesterase (AChE), chlorpropham acts primarily as a microtubule inhibitor (Veras et al., 2024; Lee et al., 2020; Campbell et al., 2010). Yet both chemicals led to enrichment of GO terms associated with motor proteins and cytoskeletal function, with carbofuran showing enrichment for pathways linked to concentric hypertrophic cardiomyopathy, a phenotype previously linked to motor protein disruption (Herron et al., 2008; Hein et al., 2000). Structural similarity (two carbamates) did not result in mechanistic equivalence at the molecular target level but did converge on disruption of the cytoskeleton. This bodes well for predictive toxicology whereby a structurally rich and diverse database of vertebrate bioactivity should identify the hazard liability of new structures, ideally without animal testing.

Notably, chlorpropham was unique in enriching for functional terms related to *myc* (also known as *mych* in zebrafish), a transcription factor critical for regulating cell growth, which was not observed with any other compound in this study (Hong et al., 2008). The cytoskeletal convergence extended beyond carbamates to include chemicals from distinct chemical subclasses and intended targets: chlordane, tefluthrin, fenamidone, and picoxystrobin, all disrupted motor protein and cytoskeletal pathways. Comparisons with CTD data revealed that while fenamidone and picoxystrobin were associated with some cytoskeletal-related terms, none of the four showed enrichment for motor protein-related terms, a novel insight of our developmental transcriptomic approach (Supplementary Table S9). The convergence suggested that structurally diverse chemicals can induce similar downstream phenotypes through varied mechanisms, possibly involving calcium signaling, oxidative stress, or energy disruption (Hepler, 2016; Myers and Casanova, 2008). Agrichemical developmental toxicity is complex. Structurally similar compounds (like carbofuran and chlorpropham) can act through divergent molecular targets to ultimately perturb the same cellular processes, while structurally unrelated compounds can converge on similar developmental outcomes.

### 4.4 Integrated analysis reveals convergent neurodevelopment disruptions

The integration of differential gene expression analysis with coexpression network modeling revealed that agrichemical exposure predominantly perturbed neurodevelopment processes, cellular metabolism and signaling homeostasis. GO enrichment analysis identified significant disruption of neuronal development and synaptic function, including neurogenesis, neuron projection development, synaptic signaling, and dendritic organization (Supplementary Table S4; Figure 4). These findings were reinforced by coexpression modules enriched for neuroligin/neurexin interactions and monoaminergic G protein-coupled receptors, suggesting potential disruption of neurotransmission and synaptic connectivity (Liu et al., 2022; Meriney and Fanselow, 2019).

The analysis also revealed perturbation of intracellular regulatory pathways, including small GTPase activity, NTP regulation, and signaling cascades involving *foxO*, *erbB*, and insulin signaling. Structural and morphogenetic processes including cytoskeletal dynamics, motor protein function, and cell adhesion were also implicated across multiple chemicals, indicating possible effects on cell shape, polarity, and migration during critical developmental windows. Beyond neurodevelopmental pathways, module-specific enrichment for L-carnitine metabolism, carbon metabolism, and pericarditis suggested broader systemic impacts, including potential metabolic and cardiovascular effects (Virmani and Cirulli, 2022). Enrichment of the GO term *salmonella* infection in module 7 likely reflected disruption of immune and inflammatory pathways, rather than an actual infection, and may indicate broader immune signaling perturbations alongside other key biological processes. Many of the agrichemicals exerted pleiotropic effects, with neurodevelopmental disruption as the primary consequence and metabolism signaling homeostasis as secondary targets.

### 4.5 Novel insights beyond developmental transcriptomics

Our developmental zebrafish transcriptomic approach revealed insights that extended beyond existing toxicogenomic knowledge. Comparison with the CTD identified 88 overlapping GO terms confirming that our approach effectively recapitulated known biology. We also discovered 409 additional GO terms not previously linked to these chemicals in CTD. This suggested that over 56% of our identified terms were novel associations. The most significant terms related to GTPase activator activity and nervous system development emerged as prominent parent categories in our data but were underrepresented in CTD, suggesting that our approach offered complementary insights, particularly regarding neurodevelopmental pathways.

The divergence between our findings and CTD was pronounced in biological emphasis: while CTD was enriched for terms related to immune signaling and BMP pathways, our dataset was dominated by effects on cytoskeletal organization and nervous system development. This divergence reflects the unique sensitivity of the developing zebrafish model to capture early transcriptional events that precede morphological changes, as well as the temporal specificity of our 48 hpf sampling timepoint. The absence of nervous system terms in the overlap with CTD suggested that many neurodevelopmental effects captured in our dataset represent previously uncharacterized mechanisms of agrichemical toxicity. The developmental zebrafish would seem to have adequate capacity to reveal both mechanistic diversity and biological convergence. We believe there is added value to a standardized developmental transcriptomic platform for identifying mechanistic pathways that would likely be missed with traditional toxicological approaches.

### 4.6 Implications for chemical classifications and risk assessment

Our findings challenge conventional approaches to agrichemical classification and risk assessment that rely primarily on intended target mechanisms. The observation that structurally similar compounds produced divergent transcriptional responses, while structurally dissimilar compounds could perturb shared developmental and transcriptional responses, demonstrates the limitations of current classification schemes. For example, while carbamate insecticides are traditionally grouped by their cholinesterase inhibition mechanism, this fails to capture the broader spectrum of developmental effects observed across different carbamates (Ahmad et al., 2024). Similarly, potency variation within a class can be substantial, as observed previously with different cholinesterase inhibitors (Risher et al., 1987).

A reductionist approach of broad intended target classifications hinders research like ours that can inform the development of more sophisticated classification systems based on nuanced transcriptional changes and biological outcomes.

## 5 Conclusion

We demonstrated the value of phenotypically anchored transcriptomic analysis in developing zebrafish to more comprehensively identify the mechanisms of agrichemical toxicity. We observed complex patterns where structurally similar compounds exhibited divergent transcriptional responses, while unrelated chemicals modulated shared biological pathways, particularly those involved in neurodevelopment and cytoskeletal organization.

Our findings highlight the utility of comparative transcriptomics to both validate known toxicological mechanisms and to uncover previously uncharacterized biological responses. Comparison with existing toxicogenomic databases revealed that our developmental transcriptomic approach identified 409 novel chemical-GO term associations, representing a 56% expansion beyond current knowledge. The predominant enrichment of neurodevelopmental and cytoskeletal terms in our dataset, compared to immune and BMP signaling pathways in existing databases, underscored the unique sensitivity of early developmental stages to chemical perturbation.

These findings have significant implications for chemical risk assessment. The convergence of diverse agrichemicals on shared developmental pathways suggested that current approaches underestimate developmental hazard. Our results support pathway-based chemical classification to identify chemicals with similar biological effects regardless of structural similarity. Our study provides a foundation for more mechanistically informed approaches to chemical safety assessment and being in a fish model, is a significant step toward elimination of mammalian testing while improving hazard detection.

## Data Availability

The datasets presented in this study can be found in online repositories. The names of the repository/repositories and accession number(s) can be found in the article/Supplementary Material.
