# Supplementary material for: Phenotypically anchored transcriptomics across diverse agrichemicals reveals conserved pathways and unique gene expression signatures in zebrafish
Source: Front Toxicol. 2025 Oct 17;7:1675060. doi: 10.3389/ftox.2025.1675060 (PMC12575134; doi:10.3389/ftox.2025.1675060)
Supplement: Supplementary file 10 [file DataSheet1.docx]

Supplementary Material


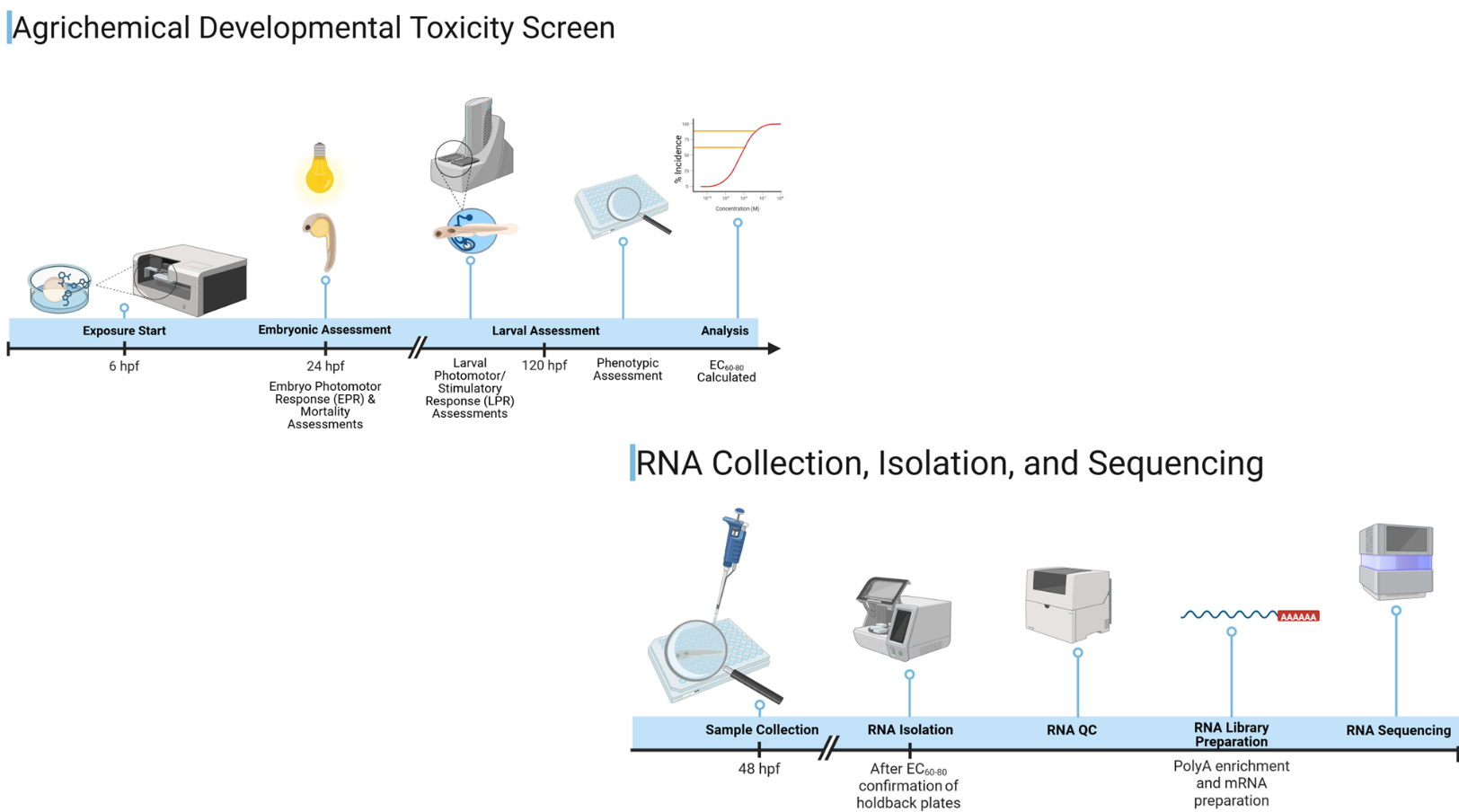


Supplementary Figure S1. Experimental overview of the developmental toxicity screen and RNA extraction and sequencing paradigm.


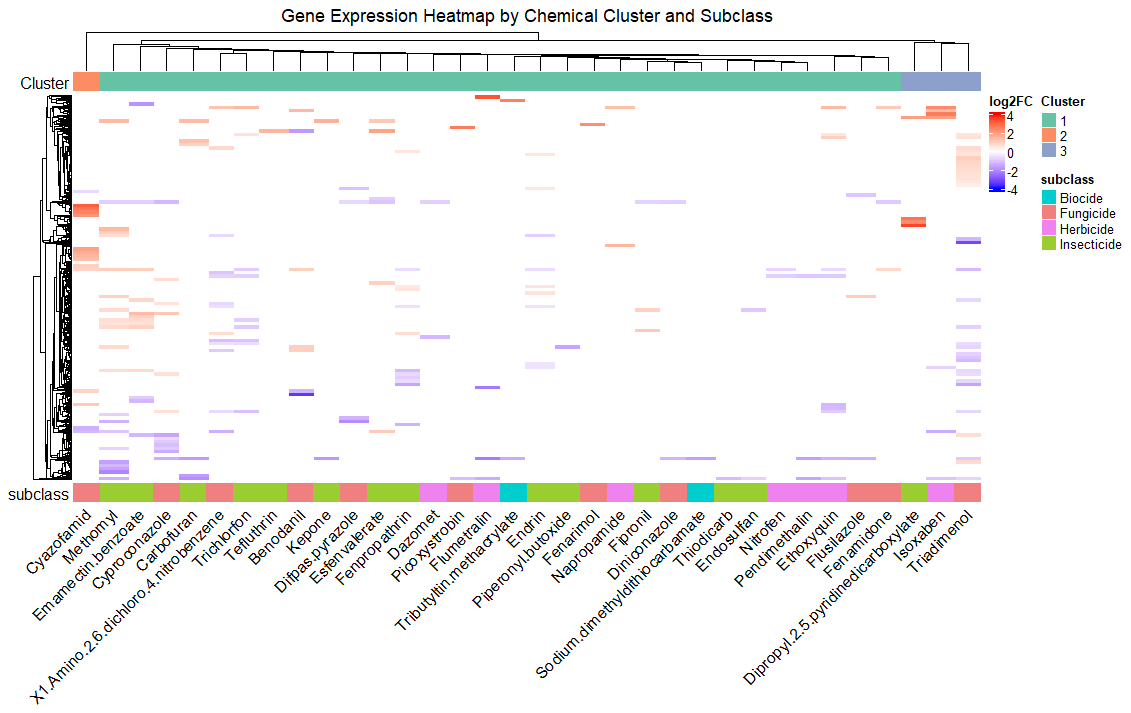


**Supplementary Figure S2.** Heatmap of all differentially expressed genes (DEGs) identified across the 34 chemicals within **Figure 3** heatmap Cluster 1. Rows represented 3,208 unique DEGs, no genes were shared across all chemicals, though some overlap occurs among subsets. Chemical subclass annotations are shown at the bottom, and cluster assignments derived from hierarchical clustering are shown at the top. Chemicals were clustered using a custom R script based on dendrogram height differences to define major clusters.

**
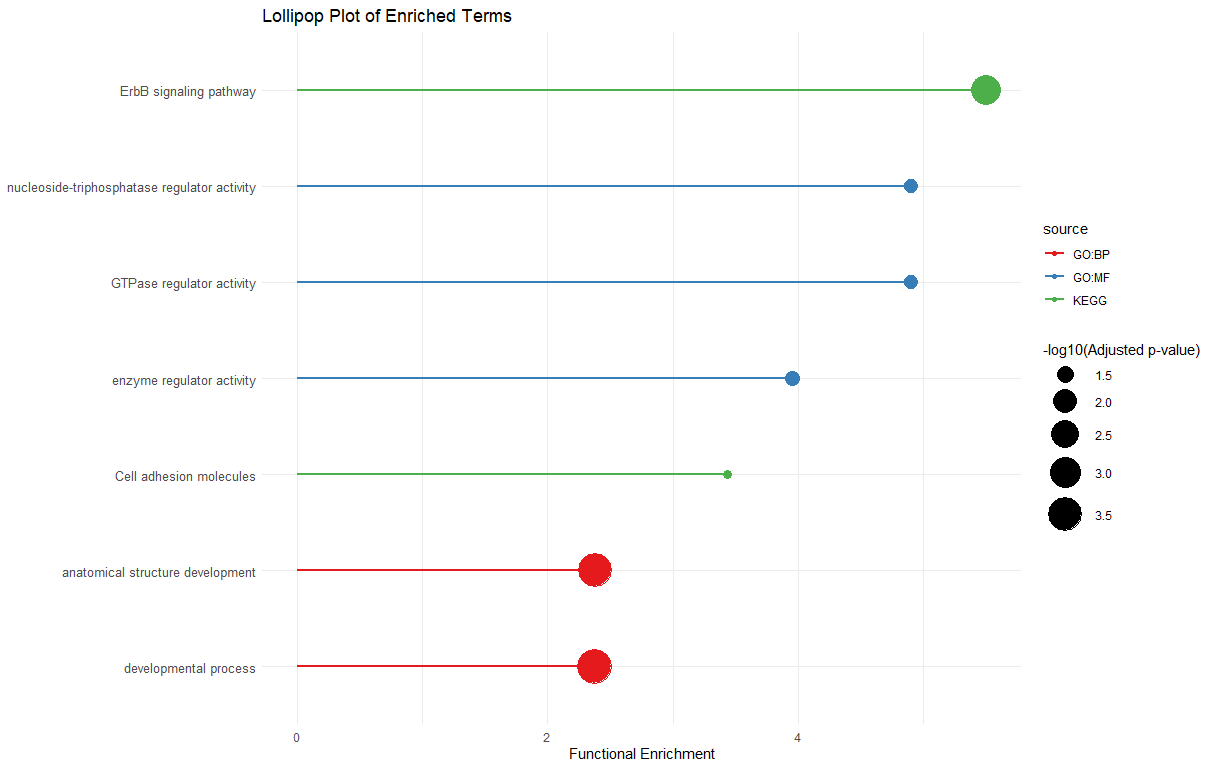
**

**Supplementary Figure S3.** Lollipop plot of Cluster 3 functional enrichment of biological terms associated with the 479 overlapping DEGs across all four agrichemicals. *ErbB* signaling was the most highly enriched, which include EGFR receptors that signal through kinases to regulate many cell activities (proliferation, migration, differentiation, apoptosis).
